# Retrospectively synchronized time‐resolved ventricular cine images from 2D real‐time exercise cardiac magnetic resonance imaging

**DOI:** 10.1111/cpf.70027

**Published:** 2025-09-03

**Authors:** Julius Åkesson, Jonathan Edlund, Katarina Steding‐Ehrenborg, Einar Heiberg

**Affiliations:** ^1^ Clinical Physiology, Department of Clinical Sciences Lund Lund University, Skåne University Hospital Lund Sweden; ^2^ Department of Biomedical Engineering, Faculty of Engineering Lund University Lund Sweden

**Keywords:** cardiovascular, free‐breathing, gating, image‐space, respiration, temporal, volumes

## Abstract

Breath‐hold ECG‐gated cardiovascular magnetic resonance (CMR) imaging is challenging during exercise due to motion, ECG‐problems, and lengthy scans. To facilitate time‐resolved volumetric measures from exercise‐CMR, we aimed to develop a method for constructing time‐resolved ventricular cines from real‐time free‐breathing exercise‐CMR. Time‐resolved ventricular cines were semi‐automatically constructed from real‐time exercise‐CMR by identifying end‐expiratory timeframes, identifying one R‐R interval within these timeframes, and synchronizing R‐R intervals across slice positions. To investigate utility, ECG‐gated rest CMR and real‐time exercise‐CMR images were collected from ten healthy volunteers and ten heart failure patients. The consistency of the left ventricular mass (LVM) was assessed between rest and exercise at end diastole (ED), mid systole (MS), end systole (ES), and early rapid filling (ERF). When comparing LVM between rest and exercise for healthy volunteers, bias ± SD was 1.5 ± 2.7 g at ED, 0.9 ± 3.3 g at MS, 1.3 ± 3.3 g at ES, and 1.2 ± 3.3 g at ERF. When comparing LVM between rest and exercise for heart failure patients, bias ± SD was 1.6 ± 2.8 g at ED, 1.0 ± 2.7 g at MS, 1.5 ± 2.6 g at ES, and 1.6 ± 2.5 g at ERF. The bias ± SD between ED and ES in standard rest images was 0.0 ± 0.7 g for healthy volunteers, and 0.0 ± 0.5 g for heart failure patients. The method for constructing time‐resolved ventricular cines from real‐time exercise‐CMR demonstrated utility for time‐resolved volumetric measurements in healthy volunteers and heart failure patients.

## 1 BACKGROUND

Cardiovascular Magnetic Resonance (CMR) imaging during exercise is associated with challenges including motion, issues with ECG‐gating, and high heart rates. Motion arises from exercise‐induced bulk movement as well as free‐breathing. Free‐breathing can cause displacement of the heart in both the in‐plane and through‐plane directions, and affect ventricular filling (Claessen et al., 2014). For the accuracy of volumetric assessments from CMR, it is therefore necessary for the image volume at each time point to consist of slices that are acquired at similar respiratory and cardiac states. In standard clinical CMR at rest, this is achieved through breath‐holding and ECG‐gating. However, during free‐breathing exercise, the quality of an ECG signal is compromised by the magneto‐hydrodynamic effect (Craven et al., 2020; Gerche et al., 2013) and motion (Craven et al., 2020; Ruijsink et al., 2017). Consequently, 2D real‐time imaging is the preferred method to facilitate imaging during exercise (Trankle et al., 2022). To utilize 2D real‐time CMR for volumetric assessments, respiratory and cardiac states need to be accurately synchronized across slice positions. This requires accurate methods for gating. However, due to the lack of external gating signals in real‐time exercise CMR, gating must be carried out directly in either k‐space or image space.

Image‐based gating of respiratory and cardiac states in CMR has been carried out in several previous studies. For image‐based respiratory gating, manifold learning for quantifying respiratory motion has been widely used (Edlund et al., 2022; Kerfoot et al., 2018; Ruijsink et al., 2017; Usman et al., 2015). For image‐based cardiac gating, several previous studies have carried out the identification of end‐diastolic and end‐systolic timeframes as a manual task (Edlund et al., 2022; Gerche et al., 2013). However, for some use cases, including the estimation of noninvasive pressure‐volume loops (Seemann et al., 2019), time‐resolved CMR images covering the entire cardiac cycle are needed. For obtaining time‐resolved CMR image volumes, previous studies have carried out cardiac gating and binning of data from multiple R‐R intervals, and have for this purpose utilized image‐based gating signals from manifold learning (Usman et al., 2015), temporal frequency spectrum analysis (Ruijsink et al., 2017), and deep learning‐based segmentation of the left ventricular (LV) blood pool (Kerfoot et al., 2018).

Retrospective binning of cardiac phases without an external cardiac signal requires assumptions regarding the appearance of image features for each cardiac state. One can however argue that this is suboptimal for exercise imaging where the cardio‐mechanical response to exercise may differ greatly between different pathologies and individuals. To allow more accurate measures of exercise‐induced cardiac mechanics without the need for extensive physiological assumptions, we instead preserved the true temporal coherence between timeframes by using consecutively acquired real‐time timeframes when constructing time‐resolved cines.

The aims of this study were to: (1) develop semi‐automated software for retrospectively synchronizing time‐resolved ventricular cine images from 2D real‐time exercise data while preserving consecutively acquired timeframes; and (2) investigate its utility for time‐resolved measures of cardiac function during exercise for both healthy volunteers and heart failure patients.

## 2 METHODS

### 2.1 Data acquisition

All imaging was performed on a Siemens 1.5 T MAGNETOM Aera scanner (Siemens Healthcare, Erlangen, Germany). In‐vivo data were collected in 10 healthy volunteers and 10 heart failure patients during rest and exercise. The heart failure patients were included in a previous study with the only inclusion criterion being an existing clinical diagnosis of heart failure. They were excluded if they were unable to carry out MR scanning or use the ergometer. Breath‐hold rest imaging was carried out using a Siemens product TrueFISP balanced steady‐state free precession (bSSFP) sequence for ECG‐gated segmented cine acquisition and a built‐in product reconstruction for cartesian data. Real‐time exercise imaging was also carried out using a Siemens product TrueFISP bSSFP sequence, but with a radial read‐out trajectory, and the reconstruction for this used GRAPPA factor 3 with a 5/8 partial Fourier factor. The reconstructed temporal resolution was 35–37 ms, the acquired spatial resolution 1.9 × 2.8 mm, and slices were imaged sequentially from apex to base, requiring 14–17 slice positions to cover the heart. For the healthy subjects, 800 timeframes were collected in each slice position, and for heart‐failure patients, 250 timeframes were collected based on the findings of (Edlund et al., 2022). All exercise imaging was carried out during ongoing exercise at a moderate level using a supine bicycle ergometer (Lode, Groningen, Netherlands). For healthy subjects, moderate exercise was defined as approximately 60% of the estimated maximum heart rate (220 beats per minute minus the subject age), as given from pulse oximeter measurements or ECG, depending on where the best signal was acquired. For the heart failure patients, the moderate exercise level was instead based on comparing the load on the bicycle ergometer with the load determined as moderate during an exercise test carried out before the scan. The exercise test determined the maximal power produced when cycling upright, and the maximal power during supine exercise was determined as 60% of this value based on (Gerche et al., 2013). The power produced at moderate exercise was in turn determined as roughly half of this value.

### 2.2 Algorithm for synchronization

**FIGURE 1:**
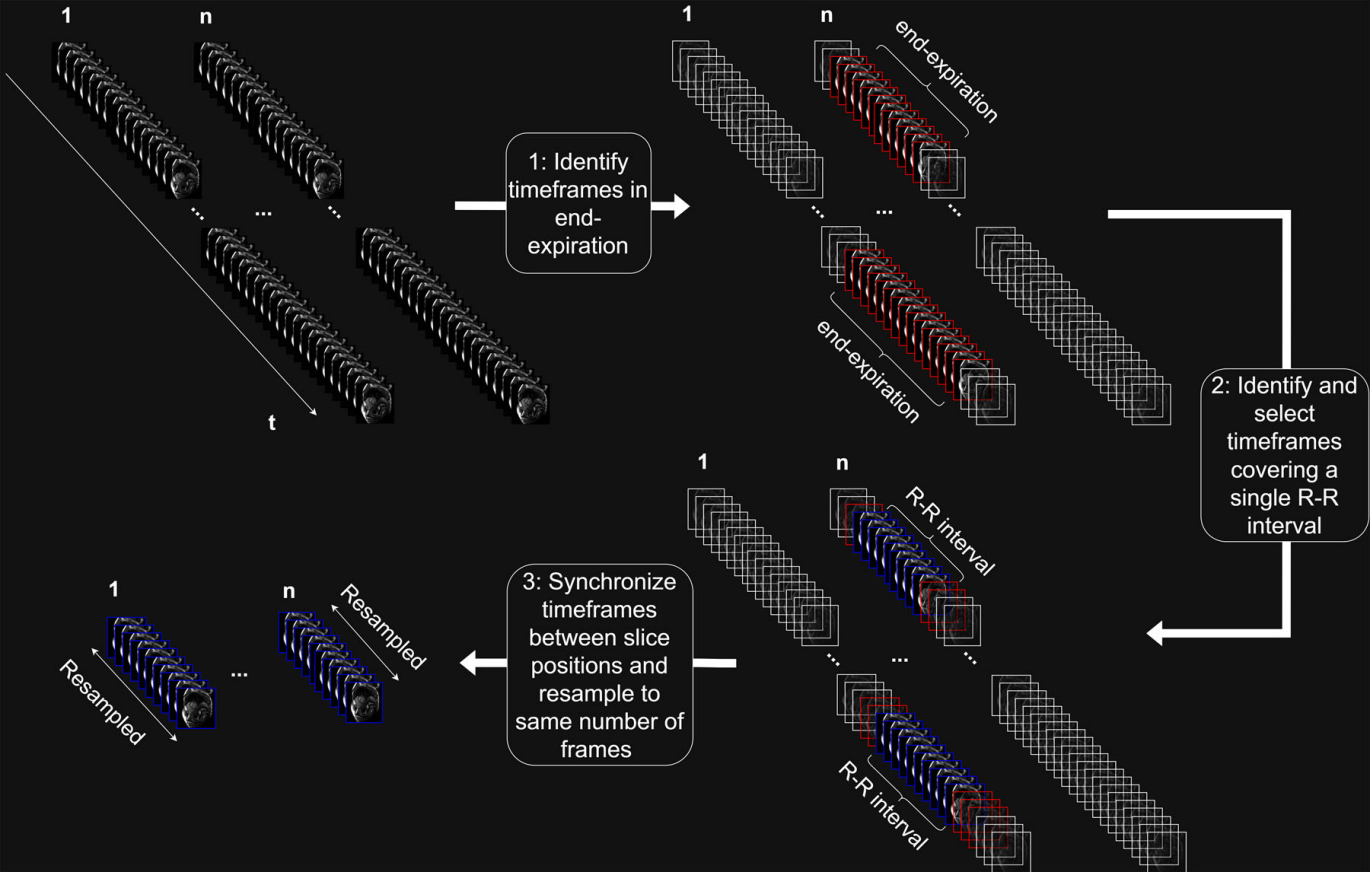
The main steps involved in constructing single‐cycle cines from multiple‐heartbeat real‐time images acquired over **n** slice positions. In the first step, timeframes are identified in the specific respiratory state of interest, which in this study was end‐expiration. In the second step, timeframes within the set of selected timeframes that corresponded to a single R‐R interval were selected. In the third step, the R‐R intervals were synchronized between slice positions and resampled to the same number of timeframes.

**FIGURE 2:**
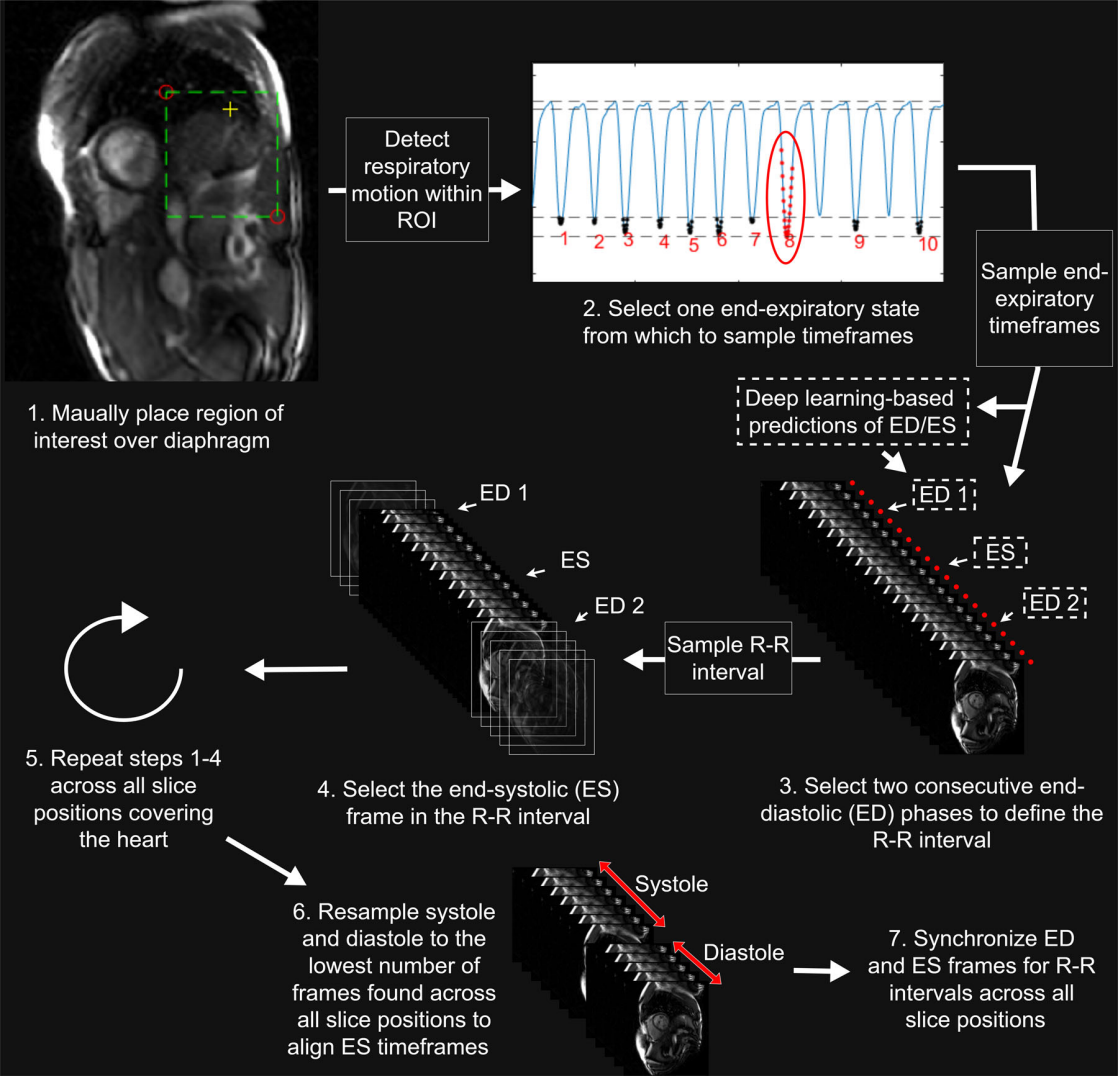
Schematic overview of the proposed semi‐automated algorithm for retrospective synchronization of time‐resolved cine images. The process included: (1) manually placing a region of interest over the diaphragm in each real‐time image series; (2) manually selecting one end‐expiratory state from which to sample timeframes (red dots), with the option to generate deep learning‐based predictions of end‐diastolic (ED) and end‐systolic (ES) phases within these timeframes; (3) manually selecting two consecutive ED phases to define the R‐R interval; (4) manually selecting the ES frame in the selected R‐R interval to allow alignment of ES across slice positions; (5) repeating steps 1‐4 across all slice positions; (6) resampling systolic and diastolic intervals separately to the lowest number of systolic and diastolic frames found across all slice positions, to allow alignment of timeframes across the spatial dimension; and finally (7) synchronize ED and ES timeframes for all R‐R intervals across all slice positions.

The task of retrospectively synchronizing time‐resolved ventricular cines from a set of 2D real‐time exercise‐CMR images was done by (Figure 1): (1) identifying and selecting timeframes near a specific respiratory state (for example end‐expiration), also known as respiratory gating; (2) identifying and selecting consecutively acquired timeframes covering a single cardiac cycle, also known as cardiac gating; and (3) synchronizing timeframes covering full cardiac cycles across slice positions after resampling these to the same number of timeframes. A semi‐automated software for carrying out each of these steps was implemented in MATLAB R2022a and added as a software plugin for *Segment* (Heiberg et al., 2010). A schematic overview of the algorithm can be seen in Figure 2, and below follows a detailed description of how each step of the algorithm was performed.

#### 2.2.1 Respiratory gating

Image‐based gating of respiratory states in real‐time images has been successfully performed using manifold learning in previous work (Kerfoot et al., 2018; Ruijsink et al., 2017; Usman et al., 2015), and we therefore also employed this method. The specific implementation used had also been previously validated for respiratory gating in real‐time exercise‐CMR (Edlund et al., 2022). In practice, the method consisted of placing a region of interest over the diaphragm (step 1 in Figure 2), followed by applying manifold learning for reducing the 2D region of interest to a 1D array that indicates the motion of the diaphragm and thus the respiratory position. The reader is referred to Edlund et al. (2022) for further details regarding this implementation (Edlund et al., 2022). Successful use of this method allowed the user to manually select one of the suggested sets of timeframes near one of the end‐expiratory peaks. This aimed to decrease the influence of respiration on the ventricular volumes as well as reducing through‐plane motion (step 2 in Figure 2). The end‐expiratory peak that had the greatest number of timeframes near its extreme was selected.

#### 2.2.2 Cardiac gating

Cardiac gating was in this study performed by identifying a set of consecutively acquired timeframes within the selected respiratory state that covered one full cardiac cycle. Due to the periodicity of cardiac motion, this was done through identifying two consecutive occurrences of the end‐diastolic (ED) phase. To semi‐automate and speed up the identification of the ED and ES phases, a method for automatic ED and ES detection was implemented. This method was applied to all timeframes selected during the respiratory gating, and was based on: (1) segmenting the left ventricle (LV) using a convolutional neural network (CNN), similarly to (Kerfoot et al., 2018; Wang et al., 2023); (2) calculating the cross‐sectional LV area in each frame from the LV segmentations; and (3) detecting ED as timeframes with a larger LV area than its three nearest frames in each direction and detecting ES as the timeframe with the smallest LV area between two ED frames. The CNN that was used to perform LV segmentation was the architecture proposed in (Bai et al., 2018). It had been previously trained on a large dataset of rest images from clinics and research, as presented in (Berggren et al., 2020).

During the assessment of the performance of the algorithm for synchronization, the step of cardiac gating was carried out fully manually, by selecting two consecutive ED frames to define the bounds of the R‐R interval in each slice position (step 3 in Figure 2). This was in part done to generate data to assess the performance of the automated ED and ES detection method.

#### 2.2.3 Synchronization

Synchronization of time‐resolved cines across slice positions was done automatically by aligning the identified ED timeframes across slice positions. The ES timeframes were used to identify systolic and diastolic timeframes (step 4 in Figure 2). Temporal down‐sampling of the systolic and diastolic intervals was carried out using a built‐in bilinear interpolation method in MATLAB. This was done to give these intervals in all slice‐positions the same number of timeframes as the corresponding intervals with the lowest total number of timeframes (step 6 in Figure 2). After this, it was possible to synchronize ED and ES timeframes for all R‐R intervals across all slice positions (step 7 in Figure 2).

### 2.3 Analyses

Retrospectively synchronized cines were created from all acquired real‐time exercise images by a physician (JE) with experience in manual ventricular delineations in both rest and exercise images. In both the rest and exercise images, left ventricular epicardial and endocardial borders were also manually delineated using the software Segment (Heiberg et al., 2010). The delineations in the exercise images were carried out at the end‐diastolic (ED), mid‐systolic (MS), end‐systolic (ES), and early rapid filling (ERF) phases to allow volumetric and LV mass (LVM) assessments across the cardiac cycle. The MS and ERF phases were selected as the two additional cardiac phases since they are the two phases with the fastest cardiac motion and thus the most substantial image blurring. This generally makes them the most challenging timeframes to delineate. Rest images were only delineated in ED and ES.

The LVM of the exercise images were at each cardiac phase compared with either the LVM of the corresponding phase from the rest images (for ED and ES) or with the ED phase of the rest images (for MS and ERF). Agreements between these measures in terms of bias and standard deviation (SD) were assessed using Bland‐Altman analysis (Bland and Altman, 1986). Bias and SD were also calculated as a percentage of the LVM at rest. Comparing LVM between two different imaging methods is an established way of comparing imaging methods, since the anatomical mass of the ventricle is constant over time, regardless of exercise or resting status. All analyses were carried out separately between healthy volunteers and heart failure patients. This was done to allow assessing any difference in method performance between the two groups. The intra‐observer variability in LVM measurements was assessed for ED and ES in the exercise images of the 10 heart failure patients (Supporting Information S1: Figure 1). For this assessment, the manual delineations were redrawn while the selections of ED and ES timeframes were reused. LV endocardial volumes throughout the cardiac cycle were also compared between rest and exercise. This was done to assess whether changes in volume agreed with what is expected from literature on the cardiac response to supine exercise assessed using CMR, that is, a decreased end‐systolic volume and an unchanged end‐diastolic volume (Beaudry et al., 2018).

Manual selections of ED and ES timeframes were compared with selections made automatically by the ED and ES detection method to assess its performance. This assessment was done for all 20 subjects in the most central midventricular slice and for the respiratory peaks selected by a physician (JE). Agreements between manual and automated selections were assessed through the mean and SD of the selected timeframe (T) difference $∆T=\;T_{\mathrm{automatic}}-T_{\mathrm{manual}}$.

All image analysis and statistics were carried out using MATLAB R2022a.

## 3 RESULTS

**FIGURE 3:**
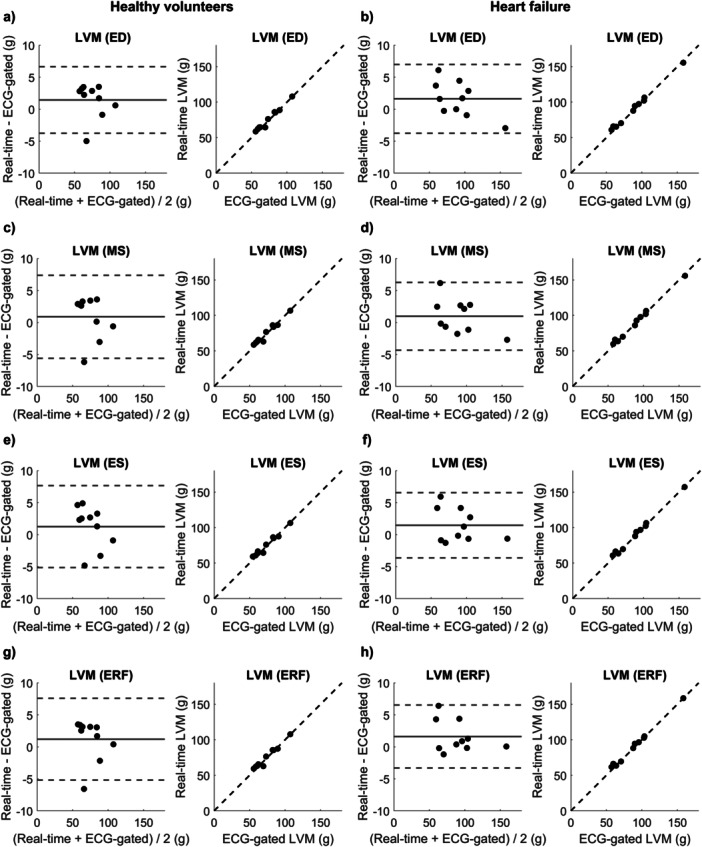
Left column: Bland‐Altman plots and correlation plots of left ventricular mass (LVM) between ECG‐gated rest and real‐time exercise images for healthy volunteers in end diastole (Figure 3a), mid systole (Figure 3c), end systole (Figure 3e), and early rapid filling (Figure 3g). Right column: Bland‐Altman plots and correlation plots of left ventricular mass (LVM) between ECG‐gated rest and real‐time exercise images for heart failure patients in end diastole (Figure 3b), mid systole (Figure 3d), end systole (Figure 3f), and early rapid filling (Figure 3h).

**FIGURE 4:**
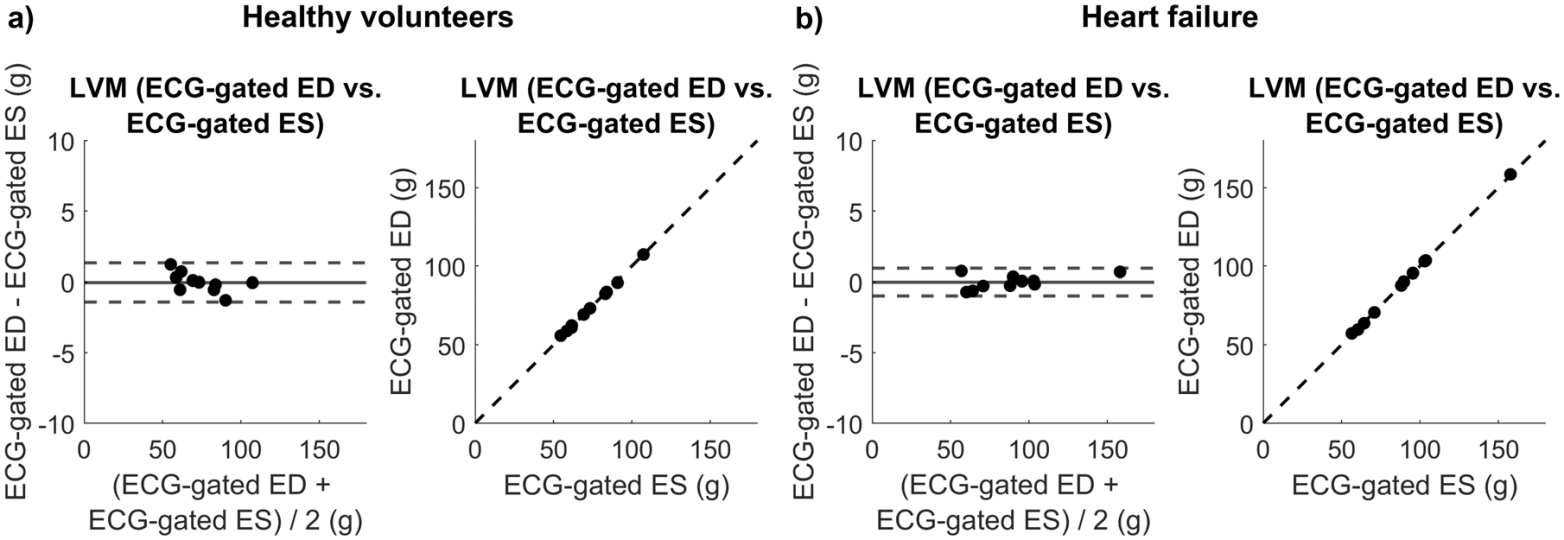
Bland‐Altman plots and correlation plots of left ventricular mass (LVM) for ECG‐gated rest images between end diastole (ED) and end‐systole (ES) for (a) healthy volunteers, and (b) heart failure patients.

When performing Bland‐Altman analysis of the LVM between rest and exercise for the healthy volunteers, bias ± SD was 1.5 ± 2.7 g (2 ± 4%) at ED (Figure 3a), 0.9 ± 3.3 g (2 ± 5%) at MS (Figure 3c), 1.3 ± 3.3 g (2 ± 5%) at ES (Figure 3e), and 1.2 ± 3.3 g (2 ± 5%) at ERF (Figure 3g). When comparing LVM between rest and exercise for heart failure patients, bias ± SD was 1.6 ± 2.8 g (3 ± 4%) at ED (Figure 3b), 1.0 ± 2.7 g (2 ± 4%) at MS (Figure 3d), 1.5 ± 2.6 g (2 ± 4%) at ES (Figure 3f), and 1.6 ± 2.5 g (2 ± 4%) at ERF (Figure 3h). For comparison, bias ± SD between ED and ES within the standard rest images was 0.0 ± 0.7 g (0 ± 1%) (Figure 4a) for healthy volunteers, and 0.0 ± 0.5 g (0 ± 1%) (Figure 4b) for heart failure patients. For the assessment of intra‐observer variability in LVM, bias ± SD between the initial and repeated measurements was −0.1 ± 2.1 g (0 ± 2%) at ED, and −0.2 ± 2.1 g (0 ± 3%) at ES (Supporting Information S1: Figure 1).

**FIGURE 5:**
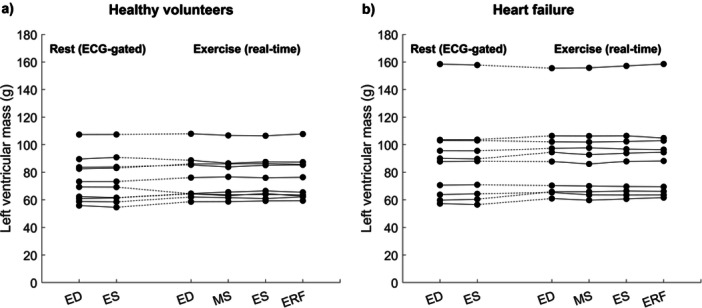
Left ventricular mass (LVM) at the end‐diastolic (ED), mid‐systolic (MS), end‐systolic (ES), and early rapid filling (ERF) phases for ECG‐gated rest images as well as real‐time exercise images for healthy volunteers (left) and heart failure patients (right).

**FIGURE 6:**
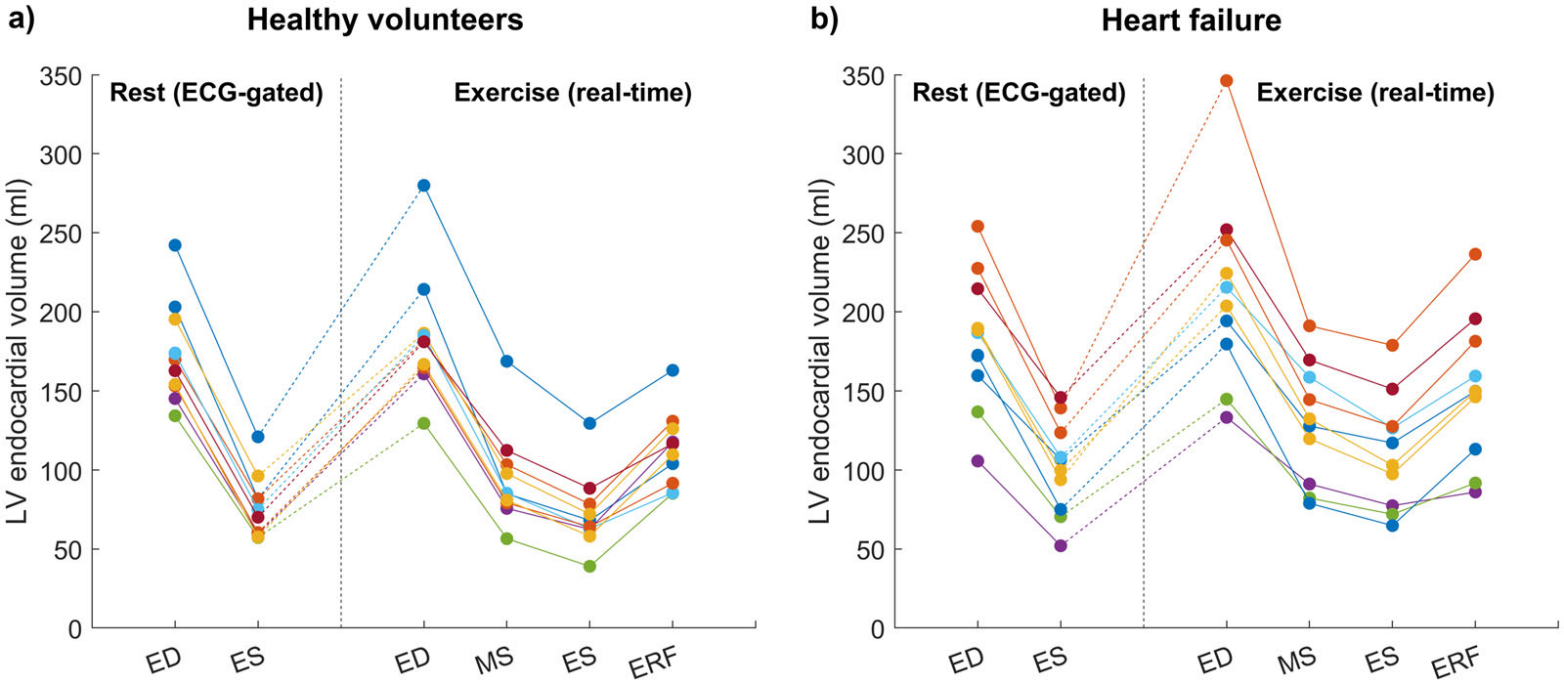
Left ventricular endocardial volumes at the end‐diastolic (ED), mid‐systolic (MS), end‐systolic (ES), and early rapid filling (ERF) phases for ECG‐gated rest images as well as real‐time exercise images for healthy volunteers (left) and heart failure patients (right). The vertical dotted lines indicate a break in the axis to separate the rest and exercise volumes.

The variability of the LVM across the cardiac cycle was low across all timepoints in exercise images, and comparable to the LVM variability across timepoints in the rest images for both healthy volunteers and heart failure patients (Figure 5). LV endocardial volumes changed over time as physiologically expected within the rest and exercise states (Figure 6). However, between rest and exercise, the ES volume did not generally decrease, and the ED volume generally changed.

In 4 of the 20 subjects, the automated ED and ES method failed to find two consecutive ED timeframes within the assessed range of timeframes. For the remaining 16 subjects, the mean and SD of the timeframe difference between automated and manual selections of ED and ES were 0.13 ± 0.91 timeframes for ED and 1.19 ± 1.60 timeframes for ES. The average number of timeframes assessed per respiratory peak was 32. The average runtime was 4 s on a laptop with a NVIDIA Quadro T1000 GPU.

## 4 DISCUSSION

We developed a semi‐automated software for retrospectively synchronizing time‐resolved ventricular cine images from 2D real‐time exercise data. Its utility was investigated for time‐resolved measures of cardiac function during exercise for both healthy volunteers and heart failure patients. The close limits of agreement in LVM between ECG‐gated rest and real‐time exercise images as well as the agreement between different time points in the exercise images indicate that the anatomical information that can be observed with standard cine imaging is preserved in retrospectively synchronized cines. The mid‐systolic and early rapid filling phases, in which the most significant motion blurring occurs, showed no significantly increased deviation in LVM compared to real‐time images or compared to the other cardiac phases. Thus, this indicates that time‐resolved volumetric measures are on par with ED and ES measures in terms of reliability. Furthermore, the intra‐observer variabilities in LVM at exercise were comparable to the inter‐method variabilities between rest and exercise.

A generally positive bias of up to 3% of the rest LVM was observed, indicating that the observer who traced the LVM overestimated the LV epicardium or underestimated the LV endocardium in the exercise images. This could be due to blurring of the images from exercise motion, breathing motion, or interpolation. With the current data, it is not possible to determine if this is due to observer bias or due to inadequate image quality.

From literature, it is known that supine exercise is associated with a decrease in left ventricular end‐systolic volume and an unchanged end‐diastolic volume in healthy subjects (Beaudry et al., 2018). The LV endocardial volume measurements between rest and exercise in healthy volunteers (Figure 6a) exhibited no general trend contradicting this. However, the measurements contained examples of increased end‐systolic volumes as well as changes in the end‐diastolic volumes between rest and exercise. This could be attributed to the low exercise intensity or to measurement inaccuracies due to bulk motion.

The temporal resolution used for real‐time imaging in this study (35–37 ms) was similar to that used in many recent real‐time exercise‐CMR studies, where the temporal resolution has been around 30–39 ms (Gerche et al., 2013; Lurz et al., 2009; Morales et al., 2022; Ruijsink et al., 2017; Trankle et al., 2022). This means that with the use of real‐time imaging with standard temporal resolution, time‐resolved volumetric measurements can be obtained using the presented method. To limit the manual delineation time, rest images were only delineated in ED and ES. This was motivated by rest imaging being a gold standard method, from which it should be possible to assume that the observed left ventricular mass is the same across time.

Since R‐R intervals were selected from time intervals *near* end‐expiratory peaks of the respiratory curve, some R‐R intervals suffered from breathing motion. This increased the influence of breathing compared to if timeframes would only be sampled from respiratory peaks. However, doing this would instead require the sharing of timeframes between heartbeats to construct one R‐R interval. This would in turn require additional physiological assumptions for ordering the timeframes which could decrease the accuracy in the representation of exercise‐induced cardiac mechanics.

The method for automatically detecting ED and ES exhibited a low bias and standard deviation on the 16 subjects where it did not fail. This indicates that the method can generate adequate initial guesses of ED and ES within just a few seconds, which can decrease the total workload of the user of the synchronization method. In three out of four failed cases, 20 or less timeframes had been selected near the respiratory peak, due to rapid breathing. The limited number of timeframes negatively influenced the method's ability to find two consecutive ED frames.

The ED and ES detection method did not function well at the basal and apical levels. The reason for this is that through‐plane motion of the LV creates complex LV morphologies and motion in these regions as well as affecting the performance of the CNN. This broke the assumption that the ED phases will intersect with peaks in the LV area. Therefore, an unsuccessful attempt to reformulate the problem of detecting ED and ES as a *classification* problem was conducted. For this, the ED and ES detection problem was formulated as classifying timeframes as either systolic or diastolic (Fiorito et al., 2018). Various recurrent neural network (RNN) architectures were tested, and the used training dataset consisted of synthetic real‐time data created from standard breath‐hold cine images. However, the resulting models exhibited suboptimal performance on the exercise data. This could be due to a discrepancy between the synthetic data used for training and the true real‐time data. A possible reason for this is that motion artifacts from exercise were not synthesized. Also, there is a wide range in cardiac morphology and motion between basal, apical, and midventricular slice positions that may also differ between rest and exercise.

## 5 LIMITATIONS

All delineations were performed by a single observer and no assessment of inter‐observer variability was conducted. The intra‐observer variability assessment was limited to ED and ES timeframes for heart failure patients. Furthermore, the observer was not blinded to the two groups of subjects, which could have influenced the magnitude of the observed difference. No fine‐tuning of the segmentation CNN for ED and ES detection was carried out to improve its segmentation ability on exercise CMR images.

## 6 FUTURE DIRECTIONS

Retrospective synchronization and construction of cines could be used to assess hemodynamics during free‐breathing or free‐breathing exercise. For example, 4D flow measurements typically require a cine for registering the phase contrast data to anatomy (Carlsson et al., 2012). A future use case could therefore be to use this method to aid analysis of 4D flow measurements during exercise, when standard segmented cine imaging cannot be used. Furthermore, noninvasive pressure‐volume loop measurements during exercise could be feasible to perform, if brachial pressure is also measured during image acquisition (Seemann et al., 2019).

The implemented deep learning‐based ED and ES phase detection method indicated the ability to speed up the process of finding midventricular ED and ES timeframes. If it is further improved for basal and apical slices, it could fully transform the process of finding ED and ES phases into an inspection of suggested timeframes instead of an active selection.

Simultaneous multi‐slice imaging allows collecting real‐time data that is already inherently synchronized across timeframes. Thus, combining our method with simultaneous multi‐slice imaging would be a very valuable tool for improving the reliability of the anatomical measures and speeding up the process of the retrospective synchronization.

## 7 CONCLUSION

A method for constructing time‐resolved ventricular cines from exercise‐CMR was developed. The results demonstrated that the method is feasible for time‐resolved volumetric measurements, both in healthy volunteers and heart failure patients.

## CONFLICT OF INTEREST STATEMENT

The authors declare no conflicts of interest.

## 1 ETHICS STATEMENTS

This study was approved by the Regional Ethical Review board in Lund, Sweden (reference number 948/2018) and the Swedish Ethical Review Authority, Sweden (reference number 2021‐05044). All participants provided written informed consent.

## Supporting information

supplementary_figure_1.

## Data Availability

The data that support the findings of this study are available on request from the corresponding author. The data are not publicly available due to privacy or ethical restrictions.
