# Supplementary figures and images for: Retrospectively synchronized time‐resolved ventricular cine images from 2D real‐time exercise cardiac magnetic resonance imaging
Source: Clin Physiol Funct Imaging. 2025 Sep 3;45(5):e70027. doi: 10.1111/cpf.70027 (PMC12406293; doi:10.1111/cpf.70027)

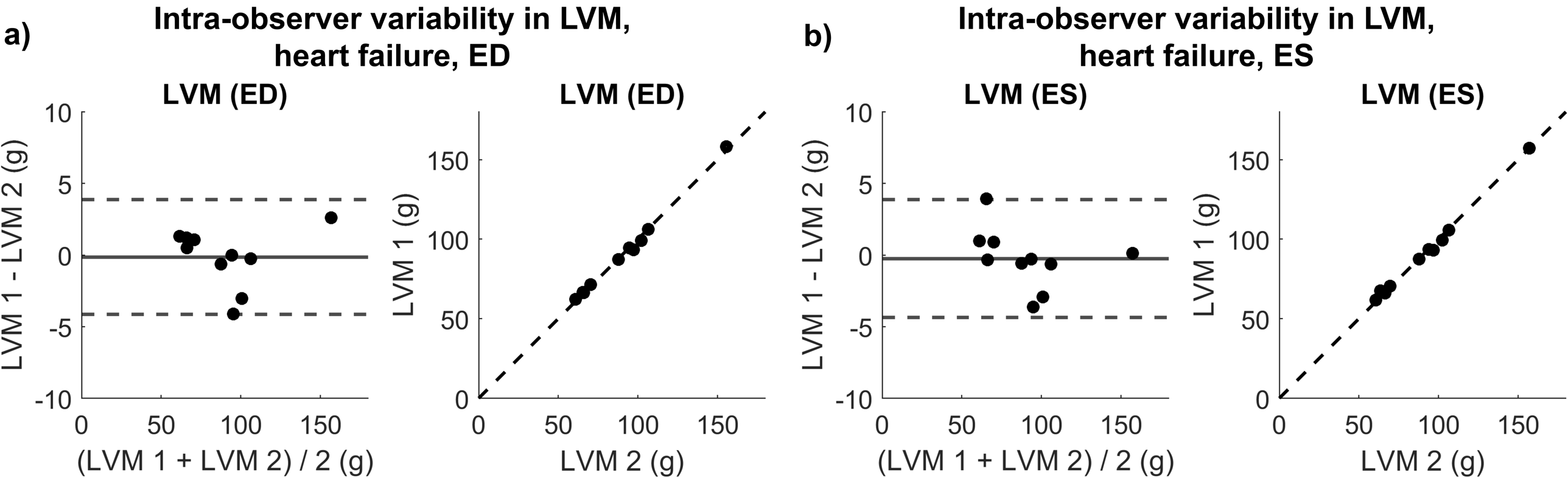

Supplement: Supplementary file 1 — supplementary_figure_1. [file CPF-45-0-s001.png]
